# Dynamics of coagulation proteins upon ICU admission and after one year of recovery from COVID-19: a preliminary study

**DOI:** 10.3389/fcimb.2024.1489936

**Published:** 2025-01-08

**Authors:** Raquel Behar-Lagares, Ana Virseda-Berdices, Óscar Martínez-González, Rafael Blancas, Marcela Homez-Guzmán, Eva Manteiga, Juan Churruca-Sarasqueta, Madian Manso-Álvarez, Ángela Algaba, Salvador Resino, Amanda Fernández-Rodríguez, María A. Jiménez-Sousa

**Affiliations:** ^1^ Unit of Viral Infection and Immunity, National Center for Microbiology (CNM), Health Institute Carlos III (ISCIII), Majadahonda, Madrid, Spain; ^2^ Centro de Investigación Biomédica en Red en Enfermedades Infecciosas (CIBERINFEC), Instituto de Salud Carlos III (ISCIII), Madrid, Spain; ^3^ Critical Care Department, Hospital Universitario del Tajo, Aranjuez, Madrid, Spain; ^4^ Universidad Alfonso X el Sabio, Villanueva de la Cañada, Madrid, Spain; ^5^ Fundación para la Investigación e Innovación Biomédica del Hospital Universitario Infanta Sofía y Hospital Universitario del Henares (FIB HUIS HHEN), San Sebastián de los Reyes, Madrid, Spain; ^6^ Critical Care Department, Hospital Universitario Infanta Cristina, Parla, Madrid, Spain; ^7^ Haematology and Haemostasis Department, University Hospital Infanta Leonor, Madrid, Spain

**Keywords:** SARS-CoV2, COVID-19 severity, biomarkers, coagulation, hospitalization, ICU

## Abstract

**Objectives:**

This study aimed to investigate the association of baseline coagulation proteins with hospitalization variables in COVID-19 patients admitted to ICU, as well as coagulation system changes after one-year post-discharge, taking into account gender-specific bias in the coagulation profile.

**Methods:**

We conducted a prospective longitudinal study on 49 ICU-admitted COVID-19 patients. Proteins were measured using a Luminex 200™. The association between coagulation protein levels and hospitalization variables was carried out by generalized linear models adjusted by the most relevant covariates.

**Results:**

At ICU admission, lower factor XII, antithrombin, and protein C levels were linked to the need for invasive mechanical ventilation (IMV) or its duration (p=0.028; p=0.047 and p=0.015, respectively). Likewise, lower factor XII, antithrombin, and prothrombin levels were associated with longer ICU length of stay (ICU LOS) (p=0.045; p=0.022; p=0.036, respectively). From baseline to the end of the follow-up, factor XII, antithrombin, prothrombin, and protein C levels notably increased in patients with longer ICU LOS. One-year post-discharge, differences were found for factor IX, aPTT, and INR. Gender-stratified analysis showed sustained alterations in males.

**Conclusions:**

Depleted specific coagulation factors on ICU admission are associated with increased severity in critically ill COVID-19 patients. Most coagulation alterations recover one-year post-discharge, except for factor IX, aPTT and INR, which remain reduced.

## 1 Introduction

Severe SARS-CoV-2 infection triggers a cascade of pathophysiological changes, including a marked inflammatory response and significant alterations in the coagulation system, leading to a hypercoagulability state involving platelet activation, coagulation cascade stimulation, and impaired fibrinolysis (de Maistre et al., 2023). Thrombosis is widely recognized as a significant contributor to organ failure among COVID-19 patients, leading to secondary complications and increased mortality (Khoshnegah et al., 2024).

The most frequent hemostatic alterations in the acute phase of COVID-19 are thrombocytosis with increased thrombin generation, followed by thrombocytopenia and elevated D-dimer levels (Ranucci et al., 2023). Critically ill COVID-19 patients show higher D-dimer and fibrin degradation product levels and longer times of prothrombin and activated partial thromboplastin (Levi et al., 2020; Tang et al., 2020). In addition, about 42% of COVID-19 patients with pneumonia in the initial waves, developed acute respiratory distress syndrome (ARDS) (Gibson et al., 2020), in which abnormal fibrin turnover occurs as a result of a procoagulant bronchoalveolar environment and impaired bronchoalveolar fibrinolysis (Livingstone et al., 2021).

However, there is limited information on coagulation protein levels and relevant hospitalization variables, especially in ICU-admitted patients, a population at higher risk for complications. Gazzaruso et al. associated low antithrombin levels with the need for mechanical ventilation and higher mortality in hospitalized COVID-19 patients (Gazzaruso et al., 2020). On the other hand, a recent meta-analysis by Khoshnegah et al. has described that lower activities of the C and S proteins might affect disease outcome being protein C considerably reduced in non-surviving patients (Khoshnegah et al., 2024). Of note, protein C level has also been described as a potential predictive marker for severe form of COVID-19 and need for ICU admission (Gharib et al., 2023). Similarly, increased levels of von Willebrand factor (vWF)-related variables have been linked to unfavorable clinical outcomes (Xu et al., 2022). Nonetheless, there are still scarce data on patients admitted to ICU and their evolution after recovery.

Besides, to elucidate whether the changes in coagulation are maintained over time, several longitudinal studies of survivors after COVID-19 have shown impaired coagulation up to 17 months after diagnosis (von Meijenfeldt et al., 2021; Fan et al., 2022; Kalaivani and Dinakar, 2022; Ranucci et al., 2023). These studies revealed increased levels of D-dimer (Fan et al., 2022), factor VIII (Fan et al., 2022), thrombin (Fan et al., 2022), vWF: Ag (Fan et al., 2022), and PAI-1 (von Meijenfeldt et al., 2021) during the follow-up compared to hospital admission. However, only one of these studies was conducted exclusively in ICU patients (Kalaivani and Dinakar, 2022), and only D-dimer was measured, which was found to be elevated both on admission and 6 months after hospital discharge. Therefore, there is a lack of information on the coagulation protein dynamics in ICU patients, especially in long-term follow-up, and their relationship with the development of future coagulopathies.

In a previous study of our group (Ceballos et al., 2021), we analyzed poorly studied coagulation proteins such as antithrombin, prothrombin, factor XI, factor XII, and factor XIII, which were differently affected at the first stages of COVID-19 depending on the degree of the disease. In this study, we described a general activation of these proteins in COVID-19 patients compared to healthy controls and a general decrease in coagulation protein levels in severe COVID-19 patients. In addition, those patients with lower levels of coagulation proteins had an increased risk of death during hospitalization (Ceballos et al., 2021).

However, it remains unknown whether baseline levels of coagulation proteins might also be associated or predictive of specific hospitalization variables during ICU stay. Additionally, there is no information on how these coagulation proteins behave in the long-term after recovery from COVID-19 in ICU patients.

In addition, it is well known that there are gender-related differences among COVID-19 patients. The risk of hospitalization, ICU admission, and mortality due to COVID-19 is higher in men than in women (Vahidy et al., 2021). Although there are some studies on the differences in coagulation profiles between male and female groups (Schwaiger et al., 2022), gender-specific differences in the coagulation status of COVID-19 patients are underrepresented in the literature.

Based on our previous study, and the lack of literature on the dynamics of these molecules, in this study, we aimed to investigate the association of baseline coagulation proteins with hospitalization variables in COVID-19 patients admitted to ICU, as well as coagulation system changes after hospital discharge (one-year post-discharge), taking into account gender-specific bias in the coagulation profile.

## 2 Methods

### 2.1 Design and study population

We performed a prospective longitudinal study in 49 COVID-19 patients with ARDS admitted to the ICU of two public hospitals in the Madrid region, the Hospital Universitario del Tajo and Hospital Universitario Infanta Cristina, between August 2020 and March 2021, who were followed up for at least one-year after discharge. The study protocol was approved by the Ethics Committee of the Institute of Health Carlos III (number CEI PI 28_2021-v3; approval date May 24^th^, 2021). Written informed consent was obtained from all patients or their authorized surrogates.

### 2.2 Clinical data

Epidemiological and clinical variables, as well as routine coagulation tests such as INR (International Normalized Ratio), aPTT (activated Partial Thromboplastin Time), and D-dimer, were collected from medical records using an electronic case report form (eCRF) built with REDCap electronic data capture tools.

All patients received enoxaparin therapy during their ICU stay. Patients in the prophylactic-dose group received 40mg/24h (<30mg/24h if renal insufficiency and 60mg/24h if weight greater than 150kg). Individuals in the therapeutic-dose category received 1mg/kg/12h of enoxaparin.

### 2.3 Samples

Plasma samples were obtained at two time points: (i) at ICU admission (baseline) and (ii) at least one-year after hospital discharge (end of follow-up). Peripheral blood samples were collected in EDTA tubes. On the same day of the extraction, samples were centrifuged to obtain plasma aliquots that were stored at -80°C until they were transferred to the National Center for Microbiology (Madrid, Spain) for subsequent analysis.

### 2.4 Coagulation proteins

Procoagulant proteins (von Willebrand factor (vWF), prothrombin, factor IX, factor XII, factor XI, factor XIII), as well as anticoagulant factors (antithrombin, protein C and protein S), were measured in plasma using Human Coagulation ProcartaPlex Panels (Invitrogen) in a Luminex 200™ analyzer (Luminex Corporation, Austin, TX, United States), according to the manufacturer’s instructions. The measured raw fluorescence intensity (FI) values (arbitrary units, a.u.) were used as previously described (Breen et al., 2015).

### 2.5 Factor and outcome variables

The following coagulation biomarkers were studied as primary factors (n=11): vWF, prothrombin, factor IX, factor XII, factor XI, factor XIII, antithrombin, protein C, protein S, aPTT, INR, and D-dimer.

The following clinical variables were studied as primary outcomes during the ICU stay (n=3): (i) need for IMV, (ii) duration of IMV, and (iii) ICU length of stay (LOS).

### 2.6 Statistical analysis

For the descriptive study, continuous variables were summarized as median and interquartile range and categorical as frequency and percentage. Significant differences between dependent groups were calculated using the Wilcoxon signed ranks test for continuous variables and the McNemar test for categorical ones.

We used generalized linear models (GLMs) to study the association between coagulation biomarkers (independent variables) and clinical outcomes (dependent variables). Firstly, we analyzed the association at baseline and the end of follow-up (cross-sectional comparisons). For this, GLMs with gamma distribution were used for continuous dependent variables, and GLMs with a binomial distribution for categorical dependent variables. Multivariate GLMs were performed to adjust for the most relevant covariates. At baseline, GLMs were adjusted by age, gender, enoxaparin dosage during ICU stay, and GOT (as an indicator of liver function). At the end of follow-up, GLMs were adjusted by age, gender, baseline GOT, follow-up time, and whether they were treated with some anticoagulant therapy after hospital discharge. Secondly, we analyzed the association between the change in coagulation protein levels from baseline to the end of follow-up (follow-up/baseline ratio) and clinical outcomes at the ICU. These GLMs were adjusted by age, gender, baseline GOT, follow-up time, enoxaparin dosage during ICU stay, and whether they needed anticoagulant treatment after discharge. Sex-related differences were explored for significant results by stratifying the analysis for males and females.

The predictive performance of each baseline model in discriminating the need for IMV was evaluated with the area under receiver operating characteristic curve (AUROC). AUROC reflects the model accuracy, with 0.70 – 0.80 representing acceptable accuracy, 0.80 – 0.90 reflecting excellent accuracy, and 0.90 - 1 being outstanding.

Statistical software R (v 4.3.0) (www.r-project.org) was used for all statistical analyses.

## 3 Results

### 3.1 Patient characteristics

At ICU admission (baseline), the median age was 60 years, 71.4% were male, and a high proportion of the study population was Caucasian (81.6%). Arterial hypertension, obesity, and diabetes were found in more than a third of the study population. Regarding the liver function biomarkers, GOT and GPT were above the normal range in 45.8% and 64.6% of patients, respectively (**Table 1**). During ICU hospitalization (**Table 2**), the mean ICU LOS was 11 days. High-flow nasal cannulas and IMV were used in 79.6% and 73.5%, respectively, with the median duration of IMV being 14 days. Almost 45% required prone positioning. At least one-year after hospital discharge (end of the follow-up), we found an improvement in liver function biomarkers (GOT and GPT) compared to baseline (**Table 1**). The median time from baseline to the end of follow-up was 13.8 months (12.5-15.6). “Neither diabetes nor obesity were significant between the study groups.”

**Table 1:** Clinical and epidemiological characteristics of patients at ICU admission (baseline) and at least one year after hospital discharge (end of follow-up).

Characteristics	Baseline	End of follow-up	p-value
**No.**	49	49	
**Age (years)**	60.0 (54.0-69.0)		
**Gender (Male)**	35/49 (71.4%)		
**BMI (kg/m^2^)**	30.9 (26.3- 35.2)	31.67 (28.1, 35.6)	0.197
Ethnicity			
Caucasian	40/49 (81.6%)		
Hispanic	6/49 (12.2%)		
Arabian	2/49 (4.1%)		
Unknown	1/49 (2.0%)		
Smoker status (n=49)			
Ex-smoker	15/49 (30.6%)		
Smoker	1/49(2.3%)		
Arterial hypertension	21/49 (42.9%)	24/49 (49.0%)	0.248
Obesity (BMI>30)	27/49 (55.1%)	30/49 (61.2%)	0.579
Diabetes	17/49 (37.4%)	20/49 (40.8%)	0.248
Hepatic Function			
GOT (UI/L)	37.5 (29.0-53.8)	20.5 (17.0-23.0)	**<0.001**
>40 UI/L	22/48 (45.8%)	3/42 (7.1%)	**<0.001**
GPT (UI/L)	45.5(34.0-63.3)	25.0 (17.0-31.0)	**<0.001**
>40 UI/L	31/48 (64.6%)	3/42 (7.1%)	**<0.001**
Therapy			
AIIRA (n=48)	7/48 (14.6%)	9/49 (18.4%)	0.062
ACE (n=48)	7/48 (14.6%)	10/47 (21.3%)	0.371
Anticoagulant therapy	49/49 (100%)	9/49 (18.4%)	

Data were summarized using standard descriptive statistics: median (interquartile range) for continuous variables and count (percentage) for categorical variables. Differences between groups were tested using the Wilcoxon signed ranks test for continuous variables and the McNemar test for categorical ones. Significant statistical differences are shown in bold. BMI, body mass index; ACE, angiotensin-converting enzyme inhibitors; AIIRA, angiotensin II receptor antagonists; ICU, intensive care unit; GOT, glutamic oxaloacetic transaminase; GPT, glutamic pyruvic transaminase.

**Table 2:** Characteristics of patients during ICU hospitalization.

Hospitalization variables	ICU patients
ICU length of stay (days)	11.0 (7.0-26.0)
Oxygen Therapy and Ventilator Support	
Oxygenotherapy	49/49 (100.0%)
High-flow nasal cannulas	39/49 (79.6%)
Time with a high-flow nasal cannula (days) (n=27)	3.0 (2.0-5.0)
IMV	36/49 (73.5%)
Duration of IMV (days)(n=36)	14.00 (6.0-23.0)
Prone position	22/49 (44.9%)
Treatments	
*Anticoagulant therapy*	
Therapeutic dose (enoxaparin) 1mg/kg/12h	6/49 (12.2%)
Prophylactic dose (enoxaparin) 40mg/24h	43/49 (87.8%)
*Other treatments*	
Antibiotics	48/49 (98.0%)
Azithromycin (n=48)	23/48 (47.9%)
Corticoids	46/49 (93.9%)

Data were summarized using standard descriptive statistics: median (interquartile range) for continuous variables and count (percentage) for categorical variables. ICU, intensive care unit.

### 3.2 Association between coagulation proteins and hospitalization variables

#### 3.2.1 Need for invasive mechanical ventilation

At baseline, lower levels of factor XII [aOR= 0.05, p=0.028], protein C [aOR=0.78, p=0.015], and a higher of INR [aOR= 1.18, p=0.028] were associated with the need for IMV, presenting the models an AUROC of 0,838, 0,887 and 0,797, respectively. For the change from baseline to the end of follow-up, factor XII and antithrombin significantly increased in those patients who had IMV [aOR=40.68, p=0.042; and aOR=121.75, p=0.045, respectively]. No differences in coagulation proteins were observed at the end of the follow-up (**Table 3**; **Supplementary Data 1**-**3**). With respect to gender differences, we found a significant association of the protein C and INR at baseline only in males [aOR=0.58, p=0.006; and aOR=1.30, p=0.033, respectively] (**Supplementary Data 4**).

**Table 3:** Association of coagulation proteins at ICU admission, during follow-up, and one year after discharge with the need for invasive mechanical ventilation (IMV), its duration, and ICU length of stay.

Time point	Coagulation proteins	Need for IMV		IMV duration		ICU LOS	
		aOR (95%CI)	*p*	aAMR (95%CI)	*p*	aAMR (95%CI)	*p*
**At ICU admission**	Factor XII	0.05 (0-0.72)	**0.028**	0.77 (0.4-1.48)	0.438	0.50 (0.29-0.89)	**0.022**
	Antithrombin	0.38 (0.08-1.75)	0.216	0.62 (0.39-0.97)	**0.047**	0.61 (0.38-0.97)	**0.045**
	Prothrombin	0.52 (0.1-2.65)	0.432	0.59 (0.35-1.02)	0.069	0.56 (0.33-0.95)	**0.036**
	Protein C	0.78 (0.63-0.95)	**0.015**	0.99 (0.93-1.06)	0.853	0.95 (0.9-1)	0.051
	INR	1.18 (1.04-1.34)	**0.012**	1.13 (0.07-18.26)	0.930	12.76 (0.84-193.87)	0.074
**From baseline to the end of follow-up**	Factor XII	40.68 (1.15-1437.87)	**0.042**	1.62 (0.58-4.53)	0.368	2.83 (1.2-6.68)	**0.022**
	Antithrombin	121.75 (1.1-13436.46)	**0.045**	2.17 (0.44-10.78)	0.351	6.55 (1.66-25.77)	**0.010**
	Prothrombin	10.54 (0.27-416.52)	0.209	2.76 (0.83-9.19)	0.110	4.25 (1.46-12.36)	**0.011**
	Protein C	55.39 (0.71-4337.09)	0.071	3.26 (0.65-16.38)	0.163	6.2 (1.74-22.11)	**0.008**
	aPTT	0.75 (0.54-3.73)	0.484	0.76 (0.58-0.98)	**0.046**	0.78 (0.6-1.01)	0.063
	INR	0.75 (0.02-11.41)	0.650	0.43 (0.06-2.92)	0.398	0.21 (0.05-0.84)	**0.035**
**One year after discharge**	Factor IX	1.11 (0.85-1.45)	0.432	0.90 (0.82-0.99)	**0.049**	0.93 (0.85-1.02)	0.141
	aPTT	1.03 (0.97-1.08)	0.345	0.99 (0.98-0.99)	**0.035**	0.99 (0.98-1)	0.119
	INR	1.07 (0.08-15.31)	0.958	0.41 (0.05-3.73)	0.439	0.28 (0.08-0.95)	**0.049**

**Statistics**: Data were calculated using generalized linear models (GLM) with binomial regression (for categorical variables) or gamma distribution (for continuous variables). Odds ratio, 95% confidence intervals (lower boundary: 2.5%, upper boundary: 97.5%), AMR, 95% confidence intervals (lower boundary: 2.5%, upper boundary: 97.5%), and p-value are shown for both adjusted models. GLMs were adjusted by age, gender, levels of GOT and enoxaparin dosage during ICU stay at baseline point; by age, levels of GOT, enoxaparin dosage during ICU stay and anticoagulant therapy during follow-up; and by age, levels of GOT and anticoagulant therapy at the end of follow-up. Significant associations are shown in bold. aPTT, activated partial thromboplastin time; INR, international normalized ratio; aOR, adjusted odds ratio; 95%CI, 95% of confidence interval; p, level of significance; vWF, von Willebrand factor, aAMR, adjusted AMR; INR, international normalized ratio; 95%CI, 95% of confidence interval; p, level of significance.

#### 3.2.2 IMV duration

At baseline, a decreased antithrombin level was associated with a longer duration of IMV [aAMR=0.62, p=0.047]. For the change from baseline to the end of follow-up, patients with a decrease in aPTT had a longer duration of IMV [aAMR=0.76, p=0.046). At the end of the follow-up, decreased factor IX and aPTT levels were found in patients with a higher duration of IMV at ICU stay [aAMR=0.90, p=0.049; and aOR=0.99, p=0.035, respectively] (**Table 3**; **Supplementary Data 5**-**6**). These differences remained only for men when analyses were stratified by gender (**Supplementary Data 7**).

#### 3.2.3 ICU LOS (days)

At ICU admission, a significant association of decreased levels of factor XII, antithrombin, and prothrombin with ICU LOS was found [aAMR=0.50, p=0.022; aAMR=0.61, p=0.045, and aAMR=0.56, p=0.036, respectively]. For the change from baseline to the end of follow-up, significant increase of factor XII [aAMR=2.83, p=0.022], antithrombin [aAMR=6.55, p=0.010], prothrombin [aAMR=4.25, p=0.011] and protein C [aAMR=6.2, p=0.008], and decrease in INR value [aAMR=0.21, p=0.035] were found in those patients with longer ICU LOS. One-year after discharge, INR remained decreased [aAMR=0.28, p=0.049] (**Table 3**; **Supplementary Data 8**-**9**).

After stratifying by gender, some significant associations remained for male group, such as decreased levels of factor XII at baseline [aAMR=0.42, p=0.028], the increase in the levels of antithrombin, prothrombin and protein C from baseline to the end of follow-up [aAMR=18.82, p=0.007; aAMR=7.71, and aAMR=10.67, p=0.005, respectively]. Likewise, reduction of the INR value from baseline to the end of the follow-up was only found in males [aAMR=0.05, p<0.001] (**Supplementary Data 10**).

## 4 Discussion

In this study, alterations in coagulation protein levels in ICU admission were associated with the need for IMV, longer duration of IMV, and higher ICU LOS. After discharge from the ICU, the presence and duration of IMV were linked to the dynamics of coagulation proteins, some of which remained altered more than one-year after hospital discharge. Besides, these associations were found particularly in males.

Binding of SARS-CoV-2 to angiotensin-converting enzyme 2 (ACE2) receptor triggers an increase in proinflammatory cytokines that can lead to systemic inflammation and activate the coagulation cascade promoting a prothrombotic status. This hypercoagulable state is characterized by an imbalance between procoagulant and anticoagulant factors, mainly in severe cases (Dunn et al., 2024). In our study, specifically, reduced levels of factor XII on ICU admission were associated with an increased need for IMV, with the model showing excellent predictive accuracy. Our results are consistent with previous studies where lower levels of coagulation proteins were described in most severe COVID-19 patients (Ceballos et al., 2021; Overmyer et al., 2021). In this sense, two studies have shown deficiencies in factor XII in 7% and 25.3% of COVID-19 patients (Bowles et al., 2020; Calderon-Lopez et al., 2021). Similarly, deficiency of factor XII has also been frequently observed in critically ill patients, such as those with sepsis (Bachler et al., 2019). Additionally, at ICU admission, the association of reduced protein C levels with the need for IMV and its excellent predictive performance is also in agreement with previous studies in hospitalized COVID-19 patients compared to controls (Marchetti et al., 2022; Belen Apak et al., 2023; Wojcik et al., 2023). Besides, Zhang et al. (2020) found that median activities of protein C were below the normal range in 19 ICU patients, which is consistent with our results. It has also been shown that levels of protein C and platelets were reduced in patients with ARDS (Livingstone et al., 2021), and these findings are related to a procoagulant state (Glas et al., 2013).

Moreover, we found that a decreased antithrombin level at ICU admission was associated with a longer duration. In this line, Gazzaruso et al. (2020) and Anakli et al. (2021) also showed a lower antithrombin activity in non-survivors compared to survivors in hospitalized and ICU patients, respectively. According to this, a study conducted on critically ill COVID-19 patients with ARDS also showed lower antithrombin levels in non-survivors (Joshi et al., 2021). In addition, antithrombin is known to be an endogenous inhibitor of coagulation and its levels are often decreased during other coagulopathy-related diseases in critically ill patients, such as sepsis and septic shock, which supports our findings (Anakli et al., 2021). Belen Apak et al. (2023) showed a significant reduction of antithrombin in ICU patients vs. healthy controls that was not shown in ward patients. Thus, antithrombin levels at ICU admission seem to clearly affect the clinical course of COVID-19 patients.

Levels of antithrombin and factor XII significantly increased from baseline to the end of follow-up in patients with the need for IMV. To the best of our knowledge, there are no studies investigating the dynamics of these two coagulation proteins in ICU patients more than one-year after hospital discharge, probably because they are not determined in any routine blood test. Regarding antithrombin, Fan et al. (2022) showed a lower level 12 months after diagnosis in 39 hospitalized COVID-19 patients compared to controls. This study only included 7 ICU patients (18%), unlike ours, which was carried out exclusively on ICU patients. Due to the different types of patients included in the study, this article does not necessarily contradict our results since we observed that a more pronounced increase in antithrombin and factor XII levels from baseline to the end of follow-up was observed in patients who had higher ICU LOS. As we have already mentioned, in the most severe patients, a depletion of coagulation factors and natural anticoagulants may occur. Thus, the increase in antithrombin and factor XII in our patients in the follow-up may respond to a recovery of hemostasis, reaching similar levels to those observed in the remaining patients admitted to ICU since no differences were observed in these coagulation proteins more than one-year after ICU discharge.

We also found lower levels of prothrombin in patients who required longer ICU LOS at ICU admission. This is consistent with previous studies, in which lower levels of prothrombin and other coagulation proteins has been associated with disease severity (Corneo et al., 2023). In addition, an increase in its level was found from baseline to the end of follow-up, with normalization at the end of follow-up.

Additionally, other coagulation proteins were altered, such as factor IX, which, despite showing no differences at admission, a significant decrease in its levels was observed after ICU discharge in patients with longer IMV duration, reaching significantly lower levels at the end of follow-up. Similar to our results, Zhang et al. (2020) observed normal levels of factor IX in critically ill COVID-19 patients at hospitalization. At the same time, Marchetti et al. (2022) found an increased level of factor IX in ICU and ward patients compared to healthy controls. These differences could be probably due to the differences in the compared groups, but additional studies with bigger sample sizes would be needed to confirm our results.

In addition, it should be mentioned that patients with higher INR had a higher probability of needing IMV during their ICU stay. In this regard, a meta-analysis of thirty-eight studies published by Zinellu et al. (2021) showed higher INR values in severe patients and non-survivors. This finding is consistent with our results since we assumed that the most severe patients required IMV. Furthermore, aPTT of patients who required a longer duration of IMV decreased from baseline to the end of follow-up and were shorter at the end of follow-up. With this respect, Fan et al. (2022) showed normal median levels of aPTT 16 months after COVID-19 recovery, with similar values to healthy controls. However, as far as we know, there is a lack of follow-up studies involving hospitalization variables and routine coagulation parameters in these patients. Additionally, it is essential to explore whether these residual abnormalities will affect long-term morbidity, therefore long-term monitoring of these markers would help clarify their potential association with future cardiac or thrombotic complications, among others long-term sequels and health risks related to coagulation disorders.

Regarding gender, we mainly observed these differences in coagulation protein levels in males, while not in females. Differences in the immune response between males and females may explain sex-specific responses to COVID-19 (Dunn et al., 2024). In addition, sex hormones regulate the expression of the ACE2 receptor and the cellular priming serine protease (TMPRSS2), both of which are involved in the entry of the virus into cells. Regulation by sex hormones may contribute to sex-associated differences in COVID-19 (Gebhard et al., 2020; Pagano et al., 2021). Regarding coagulation molecules, Pagano et al. showed a D-dimer level significantly higher in male patients with moderate or severe ARDS due to COVID-19 (Pagano et al., 2021). However, Saville et al. (2023) did not find sex differences in the D-dimer level in a meta-analysis of 11682 hospitalized COVID-19 patients from 10 studies. Therefore, our study provides additional insight into differences in the coagulation profile between men and women. However, further investigations would be necessary to determine the clinical significance of persistently low INR and factor IX in males. Still, a comprehensive analysis of sex differences in COVID-19 ICU patients should be explored in depth.

Several points should be considered in order to interpret our data correctly. First, it is important to consider that this is a preliminary study with a limited sample size. This may have reduced the likelihood of identifying statistical significance in certain subgroups. However, it should be noted that the longitudinal design of this study provides greater statistical power than would be possible in a cross-sectional study. Second, liver damage at ICU admission could influence coagulation protein levels; however, we included GOT transaminase as a covariate in the adjusted models to avoid the influence of liver abnormalities as a confounding factor. Third, there are few COVID-19 follow-up studies in the literature, and the proteins measured in our study are under-studied. It provides novelty, but further studies would be needed to clarify the dynamics of coagulation proteins in recovered patients. Likewise, a comparison with non-COVID-19 ICU patients with ARDS would provide valuable insights into whether the observed dysregulation is specific to the disease or not. Fourth, the follow-up duration of our study does not allow us to determine whether the effects of COVID-19 extend beyond one year after infection. Long-term monitoring of the coagulation markers studied would help clarify their potential association with future cardiac or thrombotic complications. To gain a deeper understanding of these alterations, future research must include well-characterized cohorts with a higher sample size, including functional analysis to investigate the mechanisms involved in the observed alterations. In accordance with this objective, our research group will undertake a study with an augmented sample size and an extended follow-up period, with the aim of elucidating the long-term consequences and health risks associated with coagulation disorders.

In conclusion, a depletion of coagulation factors (factor XII and prothrombin), as well as natural anticoagulants (antithrombin and protein C), was associated with a more severe COVID-19 during ICU stay. These alterations seem to return to normal values one-year after COVID-19 recovery, except for factor IX, INR and aPTT which remained reduced.

## Data Availability

Datasets are available on request: The raw data supporting the conclusions of this article will be made available by the authors, without undue reservation.
