# Supplementary material for: Dynamics of coagulation proteins upon ICU admission and after one year of recovery from COVID-19: a preliminary study
Source: Front Cell Infect Microbiol. 2025 Jan 8;14:1489936. doi: 10.3389/fcimb.2024.1489936 (PMC11751041; doi:10.3389/fcimb.2024.1489936)
Supplement: Supplementary file 1 [file DataSheet1.docx]

Supplementary Material

# Supplementary Data

**Supplementary Data 1**. Unadjusted and adjusted association of coagulation proteins at ICU admission (baseline), end of the follow-up and follow-up/baseline ratio with need of invasive mechanical ventilation (IMV).

| Time point | Coagulation proteins | Un-adjusted | | Adjusted | |
| --- | --- | --- | --- | --- | --- |
|  |  | OR (95%CI) | *p* | aOR (95%CI) | *p* |
| **Baseline** | Factor XII | 0.16 (0.03-0.95) | **0.044** | 0.05 (0-0.72) | **0.028** |
|  | Factor XI | 0.88 (0.45-1.74) | 0.723 | 0.96 (0.43-2.16) | 0.919 |
|  | Factor IX | 1.02 (0.88-1.17) | 0.838 | 1.00 (0.85-1.17) | 0.969 |
|  | Factor XIII | 0.99 (0.84-1.18) | 0.94 | 1.01 (0.83-1.22) | 0.917 |
|  | Antithrombin | 0.5 (0.14-1.75) | 0.281 | 0.38 (0.08-1.75) | 0.216 |
|  | Prothrombin | 0.91 (0.27-3.09) | 0.879 | 0.52 (0.1-2.65) | 0.432 |
|  | Protein C | 0.9 (0.79-1.03) | 0.134 | 0.78 (0.63-0.95) | **0.015** |
|  | Protein S | 0.94 (0.82-1.08) | 0.375 | 0.94 (0.8-1.11) | 0.489 |
|  | vWF | 1.02 (0.91-1.15) | 0.717 | 1.04 (0.9-1.2) | 0.594 |
|  | aPTT | 1.13 (0.91-1.41) | 0.268 | 1.24 (0.92-1.68) | 0.152 |
|  | INR | 1.03 (0.96-1.11) | 0.351 | 1.18 (1.04-1.34) | **0.012** |
|  | D-dimer | 1.00 (0.99-1.00) | 0.738 | 1.00 (0.99-1.00) | 0.421 |
| **Follow-up/baseline ratio** | Factor XII | 6.73 (0.62-73.44) | 0.118 | 40.68 (1.15-1437.87) | **0.042** |
|  | Factor XI | 1.66 (0.25-11.04) | 0.601 | 2.57 (0.27-24.9) | 0.414 |
|  | Factor IX | 1.84 (0.02-157.47) | 0.788 | 0.73 (0-134.12) | 0.905 |
|  | Factor XIII | 0.98 (0.34-2.82) | 0.966 | 1.40 (0.37-5.3) | 0.617 |
|  | Antithrombin | 12.76 (0.47-346.1) | 0.13 | 121.75 (1.1-13436.46) | **0.045** |
|  | Prothrombin | 2.02 (0.16-24.9) | 0.584 | 10.54 (0.27-416.52) | 0.209 |
|  | Protein C | 8.81 (0.29-265.61) | 0.211 | 55.39 (0.71-4337.09) | 0.071 |
|  | Protein S | 64.67 (0.3-13745.54) | 0.127 | 30.1 (0.05-18841.73) | 0.300 |
|  | vWF | 0.43 (0.04-4.33) | 0.475 | 0.17 (0.01-2.85) | 0.220 |
|  | aPTT | 0.75 (0.65-3.34) | 0.356 | 0.75 (0.54-3.73) | 0.484 |
|  | INR | 0.75 (0.06-3.32) | 0.418 | 0.75 (0.02-11.41) | 0.650 |
|  | D-dimer | 0.75 (0.98-7259.3) | 0.051 | 0.75 (0.42-2487.94) | 0.117 |
| **End of the follow up** | Factor XII | 0.86 (0.21-3.52) | 0.829 | 1.10 (0.24-5.06) | 0.900 |
|  | Factor XI | 0.97 (0.76-1.25) | 0.837 | 1.02 (0.78-1.33) | 0.901 |
|  | Factor IX | 1.16 (0.93-1.44) | 0.191 | 1.11 (0.85-1.45) | 0.432 |
|  | Factor XIII | 1 (0.87-1.15) | 0.96 | 1.07 (0.9-1.26) | 0.458 |
|  | Antithrombin | 1.36 (0.62-2.96) | 0.441 | 1.73 (0.67-4.44) | 0.256 |
|  | Prothrombin | 0.99 (0.41-2.44) | 0.99 | 1.03 (0.35-3.02) | 0.960 |
|  | Protein C | 1.02 (0.88-1.18) | 0.806 | 0.99 (0.81-1.21) | 0.890 |
|  | Protein S | 1.07 (0.9-1.27) | 0.43 | 1.06 (0.87-1.3) | 0.563 |
|  | vWF | 0.89 (0.64-1.22) | 0.463 | 0.89 (0.62-1.28) | 0.533 |
|  | aPTT | 1.02 (0.98-1.07) | 0.281 | 1.03 (0.97-1.08) | 0.345 |
|  | INR | 0.55 (0.1-3.07) | 0.498 | 1.07 (0.08-15.31) | 0.958 |
|  | D-dimer | 1.00 (0.99-1.01) | 0.22 | 1.00 (1-1.01) | 0.239 |

**Statistics**: Data were calculated by Generalized Linear Models (GLM) with a binomial distribution. Odds ratio, 95% confidence intervals (lower boundary: 2.5%, upper boundary: 97.5%), an p-value are shown, both adjusted and unadjusted, for every model. GLMs were adjusted by age, gender, levels of GOT and enoxaparin dosage during ICU stay at baseline point; by age, levels of GOT, enoxaparin dosage during ICU stay and anticoagulant therapy during follow-up, and by by age, levels of GOT and anticoagulant therapy at the end of follow-up. Significant associations are shown in bold. **Abbreviations**: aPTT, activated partial thromboplastin time; INR, international normalized ratio; OR, odds ratio; aOR, adjusted odds ratio; 95%CI, 95% of confidence interval; p, level of significance; vWF, von Willebrand factor.

**Supplementary Data 2**: Visual representation of adjusted association of coagulation proteins at ICU admission (baseline), end of the follow-up and follow-up/baseline ratio with the need for invasive mechanical ventilation (IMV). **Statistics**: data were calculated by Generalized Linear Models (GLM) with a binomial distribution. Odds ratio, 95% confidence intervals (lower boundary: 2.5%, upper boundary: 97.5%), an p-value are shown. GLMs were adjusted by age, gender, levels of GOT and enoxaparin dosage during ICU stay at baseline point; by age, levels of GOT, enoxaparin dosage during ICU stay and anticoagulant therapy during follow-up, and by age, levels of GOT and anticoagulant therapy at the end of follow-up. Significant associations are shown with an asterisk. Abbreviations: aPTT, activated partial thromboplastin time; IMV, invasive mechanical ventilation; INR, international normalized ratio; OR, odds ratio; aOR, adjusted odds ratio; 95%CI, 95% of confidence interval; p, level of significance; vWF, von Willebrand factor.


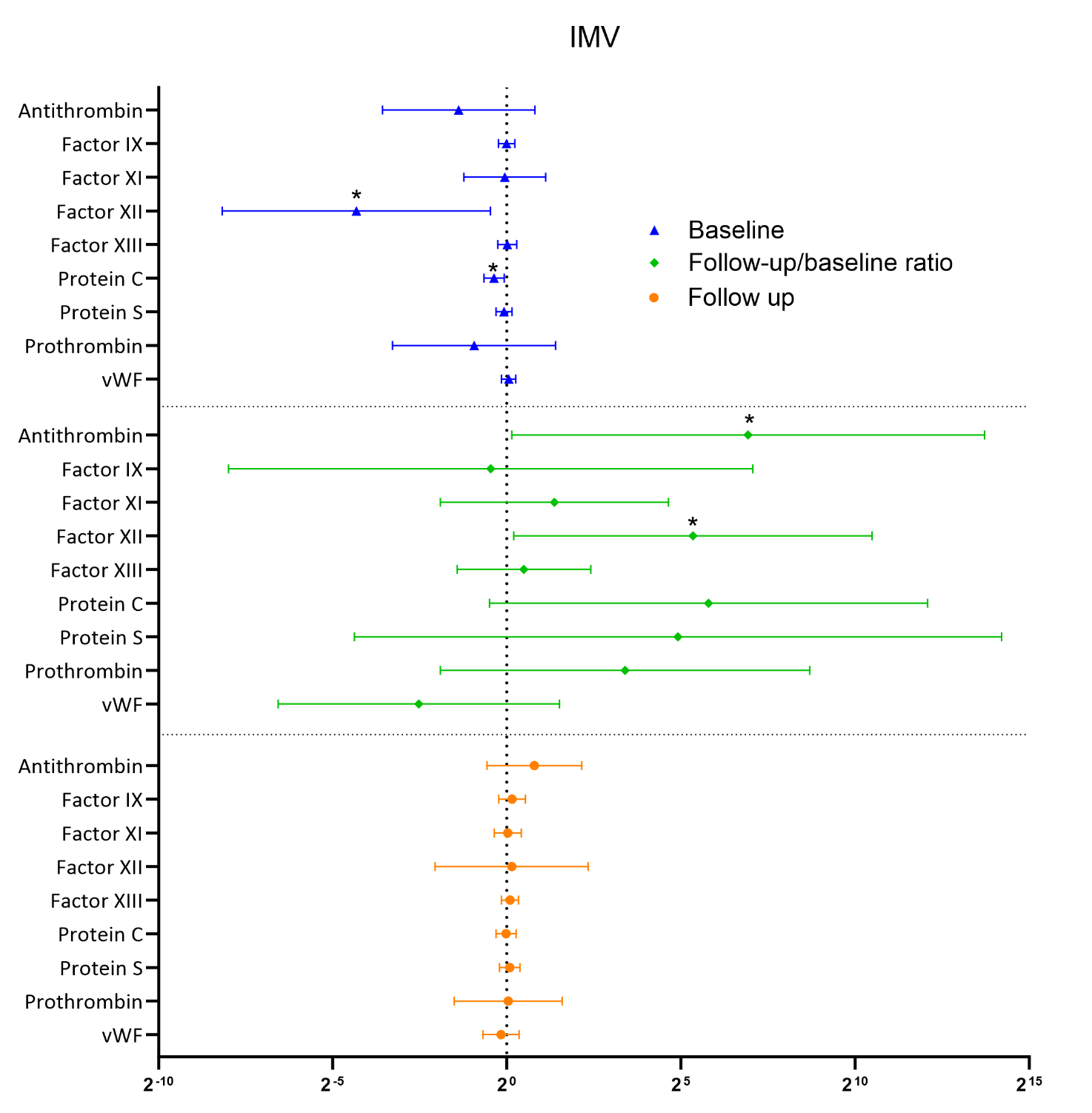


**Supplementary Data 3**. Dynamics from baseline to follow-up of antithrombin (A) and Factor XII (B). Statistics: Wilcoxon-Mann Whitney test was used to explore differences with and without IMV, both at baseline and follow-up time points (cross-sectional studies). Wilcoxon signed-rank test was used to study differences at baseline and at follow-up points, both in patients who required IMV and in those who did not. Significant differences are shown with an asterisk.


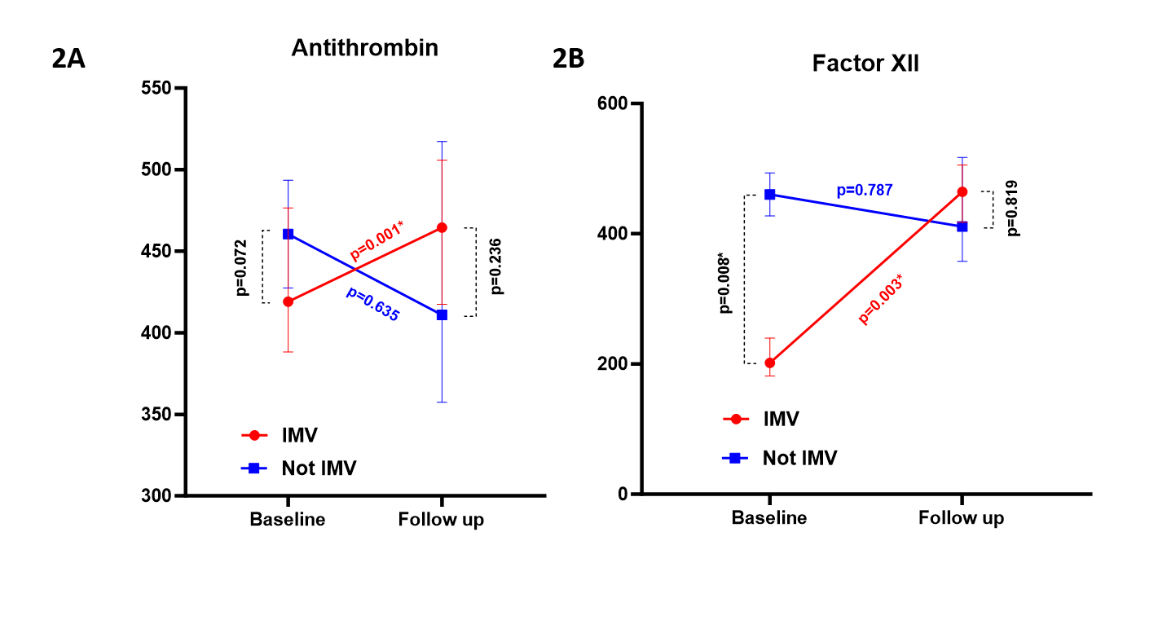


**Supplementary Data 4**. Adjusted association of significant coagulation proteins at ICU admission (baseline), end of the follow-up and follow-up/baseline ratio with the need of invasive mechanical ventilation (IMV), stratifying by gender.

| Time point | Coagulation proteins | Males | | Females | |  |
| --- | --- | --- | --- | --- | --- | --- |
|  |  | aOR (95%CI) | *p* | aOR (95%CI) | *p* |  |
| **Baseline** | Factor XII | 0.07 (0.01-1.00) | 0.050 | - | - |  |
|  | Protein C | 0.58 (0.39-0.86) | **0.006** | 1.01 (0.76-1.33) | 0.965 | |
|  | INR | 1.30 (1.02-1.65) | **0.033** | - | - |  |
| **Follow-up/baseline ratio** | Factor XII | 20.97 (0.37-1199.57) | 0.140 | - | - |  |
|  | Antithrombin | 56.53 (0.18-17737.47) | 0.169 | - | - |  |

**Statistics**: Data were calculated by Generalized Linear Models (GLM) with a binomial distribution. Odds ratio, 95% confidence intervals (lower boundary: 2.5%, upper boundary: 97.5%), an p-value are shown, both adjusted and unadjusted, for every model. GLMs were adjusted by age, gender, levels of GOT and enoxaparin dosage during ICU stay at baseline point; by age, levels of GOT, enoxaparin dosage during ICU stay and anticoagulant therapy during follow-up, and by age, levels of GOT and anticoagulant therapy at the end of follow-up. Significant associations are shown in bold. **Abbreviations**: INR, international normalized ratio; OR, odds ratio; aOR, adjusted odds ratio; 95%CI, 95% of confidence interval; p, level of significance.

**Supplementary Data 5**. Unadjusted and adjusted association of coagulation proteins at ICU admission (baseline), end of the follow-up and follow-up/baseline ratio with the duration of invasive mechanical ventilation (IMV).

| Time point | Coagulation proteins | Un-adjusted | | Adjusted | |
| --- | --- | --- | --- | --- | --- |
|  |  | AMR (95%CI) | *p* | aAMR (95%CI) | *p* |
| **Baseline** | Factor XII | 0.74 (0.42-1.3) | 0.306 | 0.77 (0.4-1.48) | 0.438 |
|  | Factor XI | 0.93 (0.72-1.21) | 0.590 | 0.9 (0.68-1.18) | 0.447 |
|  | Factor IX | 1.02 (0.95-1.08) | 0.600 | 1.02 (0.95-1.09) | 0.547 |
|  | Factor XIII | 0.98 (0.92-1.05) | 0.588 | 0.97 (0.91-1.04) | 0.377 |
|  | Antithrombin | 0.66 (0.44-1) | 0.061 | 0.62 (0.39-0.97) | **0.047** |
|  | Prothrombin | 0.58 (0.39-0.88) | **0.014** | 0.59 (0.35-1.02) | 0.069 |
|  | Protein C | 0.98 (0.93-1.03) | 0.354 | 0.99 (0.93-1.06) | 0.853 |
|  | Protein S | 1.01 (0.95-1.06) | 0.84 | 1.01 (0.95-1.07) | 0.703 |
|  | vWF | 1.03 (0.98-1.08) | 0.214 | 1.02 (0.97-1.08) | 0.468 |
|  | aPTT | 1.02 (0.97-1.08) | 0.482 | 1.01 (0.95-1.08) | 0.654 |
|  | INR | 1.85 (0.19-17.99) | 0.598 | 1.13 (0.07-18.26) | 0.93 |
|  | D-dimer | 1.00 (0.99-1.00) | 0.926 | 0.99 (0.99-1.00) | 0.528 |
| **Follow-up/baseline ratio** | Factor XII | 1.43 (0.63-3.26) | 0.401 | 1.62 (0.58-4.53) | 0.368 |
|  | Factor XI | 1.27 (0.61-2.61) | 0.525 | 1.33 (0.57-3.12) | 0.515 |
|  | Factor IX | 0.14 (0.02-0.82) | **0.036** | 0.13 (0.02-1.03) | 0.063 |
|  | Factor XIII | 1.1 (0.73-1.67) | 0.648 | 1.07 (0.64-1.77) | 0.803 |
|  | Antithrombin | 1.60 (0.43-5.95) | 0.487 | 2.17 (0.44-10.78) | 0.351 |
|  | Prothrombin | 1.56 (0.57-4.28) | 0.389 | 2.76 (0.83-9.19) | 0.110 |
|  | Protein C | 1.71 (0.47-6.25) | 0.421 | 3.26 (0.65-16.38) | 0.163 |
|  | Protein S | 0.28 (0.04-1.83) | 0.194 | 0.35 (0.04-3.41) | 0.375 |
|  | vWF | 1.02 (0.36-2.9) | 0.977 | 1.01 (0.29-3.39) | 0.997 |
|  | aPTT | 0.80 (0.63-1.01) | 0.070 | 0.76 (0.58-0.98) | **0.046** |
|  | INR | 0.95 (0.28-3.15) | 0.930 | 0.43 (0.06-2.92) | 0.398 |
|  | D-dimer | 1.12 (0.47-2.7) | 0.802 | 1.19 (0.40-3.52) | 0.759 |
| **End of the follow up** | Factor XII | 1 (0.53-1.88) | 0.999 | 1.01 (0.5-2.02) | 0.988 |
|  | Factor XI | 0.94 (0.84-1.05) | 0.259 | 0.96 (0.85-1.08) | 0.494 |
|  | Factor IX | 0.9 (0.84-0.97) | **0.010** | 0.9 (0.82-1.01) | **0.049** |
|  | Factor XIII | 1 (0.95-1.06) | 0.936 | 0.98 (0.92-1.05) | 0.614 |
|  | Antithrombin | 0.95 (0.66-1.38) | 0.803 | 0.91 (0.6-1.38) | 0.662 |
|  | Prothrombin | 0.83 (0.55-1.25) | 0.376 | 1.06 (0.65-1.72) | 0.831 |
|  | Protein C | 0.99 (0.93-1.04) | 0.621 | 1.03 (0.96-1.11) | 0.427 |
|  | Protein S | 0.94 (0.88-1) | 0.067 | 0.97 (0.89-1.05) | 0.437 |
|  | vWF | 1.06 (0.91-1.24) | 0.434 | 1.03 (0.86-1.22) | 0.765 |
|  | aPTT | 0.99 (0.98-1) | **0.029** | 0.99 (0.98-0.99) | **0.035** |
|  | INR | 1.26 (0.39-4.05) | 0.697 | 0.41 (0.05-3.73) | 0.439 |
|  | D-dimer | 0.99 (0.99-1.01) | 0.278 | 0.99 (0.99-1.00) | 0.359 |

**Statistics**: Data were calculated by Generalized Linear Models (GLM) with a gamma distribution. AMR, 95% confidence intervals (lower boundary: 2.5%, upper boundary: 97.5%), and p-value are shown, for both unadjusted and adjusted models. GLMs were adjusted by age, gender, levels of GOT and enoxaparin dosage during ICU stay at baseline point; by age, levels of GOT, enoxaparin dosage during ICU stay and anticoagulant therapy during follow-up, and by by age, levels of GOT and anticoagulant therapy at the end of follow-up. Significant associations are shown in bold. **Abbreviations**: AMR, arithmetic mean ratio; aAMR, adjusted AMR; aPTT, activated partial thromboplastin time; INR, international normalized ratio; 95%CI, 95% of confidence interval; p, level of significance; vWF, von Willebrand factor.

**Supplementary Data 6:** Visual representation of adjusted association of coagulation proteins at ICU admission (baseline), end of follow-up and follow-up/baseline ratio with the duration of invasive mechanical ventilation (IMV). Statistics**:** data were calculated by Generalized Linear Models (GLM) with a gamma distribution. AMR, 95% confidence intervals (lower boundary: 2.5%, upper boundary: 97.5%), and p-value are shown. GLMs were adjusted by age, gender, levels of GOT and enoxaparin dosage during ICU stay at baseline point; by age, levels of GOT, enoxaparin dosage during ICU stay and anticoagulant therapy during follow-up, and by by age, levels of GOT and anticoagulant therapy at the end of follow-up. Significant associations are shown with an asterisk. Abbreviations: AMR, arithmetic mean ratio; aAMR, adjusted AMR; aPTT, activated partial thromboplastin time; INR, international normalized ratio; 95%CI, 95% of confidence interval; p, level of significance; vWF, von Willebrand factor.

**
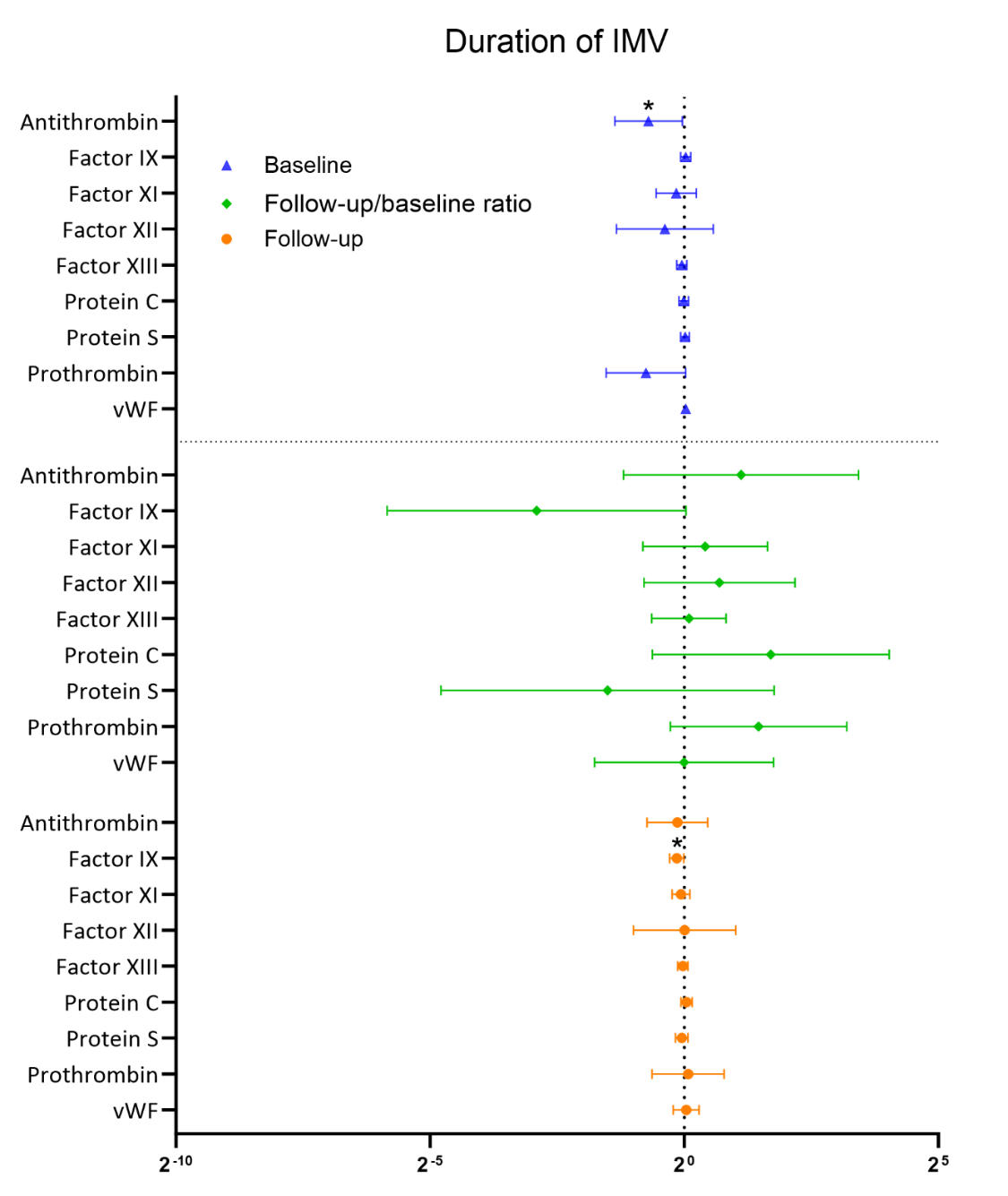
**

**Supplementary Data 7.** Association of significant coagulation factors at ICU admission (baseline), end of follow-up and follow-up/baseline ratio with the duration of invasive mechanical ventilation (IMV), stratifying by gender.

| Time point | Coagulation proteins | Males | | Females | |
| --- | --- | --- | --- | --- | --- |
|  |  | aAMR (95%CI) | *p* | aAMR (95%CI) | *p* |
| **Baseline** | Antithrombin | 0.59 (0.3-1.14) | 0.134 | 0.81 (0.42-1.55) | 0.548 |
| **Follow-up/baseline ratio** | aPTT | 0.69 (0.49-0.96) | **0.049** | 1.91 (0.9-4.06) | 0.236 |
| **End of the follow-up** | Factor IX | 0.87 (0.77-0.99) | 0.051 | 0.9 (0.83-0.98) | 0.052 |
|  | aPTT | 0.98 (0.97-1) | **0.040** | 0.99 (0.97-1.01) | 0.477 |

**Statistics**: data were calculated by Generalized Linear Models (GLM) with a gamma distribution. AMR, 95% confidence intervals (lower boundary: 2.5%, upper boundary: 97.5%), and p-value are shown, for both unadjusted and adjusted models. GLMs were adjusted by age, gender, levels of GOT and enoxaparin dosage during ICU stay at baseline point; by age, levels of GOT, enoxaparin dosage during ICU stay and anticoagulant therapy during follow-up, and by by age, levels of GOT and anticoagulant therapy at the end of follow-up. Significant associations are shown in bold. **Abbreviations**: AMR, arithmetic mean ratio; aAMR, adjusted AMR; aPTT, activated partial thromboplastin time; 95%CI, 95% of confidence interval; p, level of significance.

**Supplementary Data 8.** Unadjusted and adjusted association of coagulation proteins at ICU admission (baseline), end of the follow-up time and follow-up/baseline ratio with the ICU length of stay.

| Time point | Coagulation proteins | Un-adjusted | | Adjusted | |  |
| --- | --- | --- | --- | --- | --- | --- |
|  |  | AMR (95%CI) | *p* | aAMR (95%CI) | *p* | |
| **Baseline** | Factor XII | 0.56 (0.34-0.94) | **0.032** | 0.5 (0.29-0.89) | **0.022** | |
|  | Factor XI | 0.89 (0.69-1.15) | 0.369 | 0.88 (0.66-1.17) | 0.371 | |
|  | Factor IX | 1.02 (0.96-1.08) | 0.511 | 1.03 (0.96-1.09) | 0.430 | |
|  | Factor XIII | 0.98 (0.92-1.05) | 0.574 | 0.98 (0.91-1.05) | 0.563 | |
|  | Antithrombin | 0.66 (0.43-0.99) | 0.053 | 0.61 (0.38-0.97) | **0.045** | |
|  | Prothrombin | 0.67 (0.43-1.05) | 0.087 | 0.56 (0.33-0.95) | **0.036** | |
|  | Protein C | 0.97 (0.93-1.02) | 0.225 | 0.95 (0.9-1) | 0.051 | |
|  | Protein S | 1 (0.95-1.05) | 0.922 | 1.01 (0.95-1.06) | 0.937 | |
|  | vWF | 1.02 (0.98-1.07) | 0.351 | 1.02 (0.97-1.08) | 0.388 | |
|  | aPTT | 1.04 (0.99-1.1) | 0.153 | 1.05 (0.99-1.12) | 0.103 | |
|  | INR | 3.03 (0.31-29.46) | 0.344 | 12.76 (0.84-193.87) | 0.074 | |
|  | D-dimer | 1.00 (0.99-1.00) | 0.374 | 1.01 (0.99-1.01) | 0.103 | |
| **Follow-up/baseline ratio** | Factor XII | 2.06 (0.97-4.34) | 0.065 | 2.83 (1.2-6.68) | **0.022** | |
|  | Factor XI | 1.5 (0.75-2.96) | 0.255 | 1.58 (0.72-3.46) | 0.259 | |
|  | Factor IX | 0.23 (0.04-1.18) | 0.085 | 0.19 (0.03-1.23) | 0.088 | |
|  | Factor XIII | 1.18 (0.79-1.76) | 0.431 | 1.21 (0.75-1.97) | 0.437 | |
|  | Antithrombin | 3.01 (0.95-9.49) | 0.066 | 6.55 (1.66-25.77) | **0.010** | |
|  | Prothrombin | 1.8 (0.69-4.68) | 0.234 | 4.25 (1.46-12.36) | **0.011** | |
|  | Protein C | 2.07 (0.65-6.6) | 0.227 | 6.2 (1.74-22.11) | **0.008** | |
|  | Protein S | 0.76 (0.13-4.4) | 0.759 | 0.95 (0.1-9.09) | 0.964 | |
|  | vWF | 1.05 (0.41-2.71) | 0.923 | 0.82 (0.27-2.47) | 0.727 | |
|  | aPTT | 0.84 (0.67-1.05) | 0.126 | 0.78 (0.6-1.01) | 0.063 | |
|  | INR | 0.58 (0.23-1.44) | 0.246 | 0.21 (0.05-0.84) | **0.035** | |
|  | D-dimer | 1.36 (0.57-3.27) | 0.49 | 1.31 (0.47-3.69) | 0.613 | |
| **End of the follow up** | Factor XII | 1.13 (0.65-1.97) | 0.665 | 1.21 (0.64-2.27) | 0.555 | |
|  | Factor XI | 0.93 (0.84-1.03) | 0.158 | 0.94 (0.85-1.05) | 0.314 | |
|  | Factor IX | 0.94 (0.87-1.01) | 0.106 | 0.93 (0.85-1.02) | 0.141 | |
|  | Factor XIII | 1.01 (0.96-1.07) | 0.635 | 1.01 (0.94-1.08) | 0.825 | |
|  | Antithrombin | 1.16 (0.85-1.6) | 0.348 | 1.19 (0.82-1.73) | 0.372 | |
|  | Prothrombin | 0.88 (0.62-1.25) | 0.478 | 1.04 (0.68-1.58) | 0.856 | |
|  | Protein C | 0.99 (0.94-1.05) | 0.757 | 1.02 (0.95-1.10) | 0.626 | |
|  | Protein S | 0.96 (0.91-1.03) | 0.252 | 0.98 (0.91-1.06) | 0.618 | |
|  | vWF | 1.01 (0.89-1.15) | 0.863 | 0.99 (0.85-1.14) | 0.857 | |
|  | aPTT | 0.99 (0.98-1.00) | 0.123 | 0.99 (0.98-1.01) | 0.119 | |
|  | INR | 0.77 (0.35-1.69) | 0.519 | 0.28 (0.08-0.95) | **0.049** | |
|  | D-dimer | 1.00 (0.99-1.00) | 0.513 | 1.01 (0.99-1.01) | 0.479 | |

**Statistics**: data were calculated by Generalized Linear Models (GLM) with a gamma distribution. AMR, 95% confidence intervals (lower boundary: 2.5%, upper boundary: 97.5%), and p-value are shown, for both unadjusted and adjusted models. GLMs were adjusted by age, gender, levels of GOT and enoxaparin dosage during ICU stay at baseline point; by age, levels of GOT, enoxaparin dosage during ICU stay and anticoagulant therapy during follow-up, and by by age, levels of GOT and anticoagulant therapy at the end of follow-up. Significant associations are shown in bold. **Abbreviations**: AMR, arithmetic mean ratio; aAMR, adjusted AMR; aPTT, activated partial thromboplastin time; INR, international normalized ratio; 95%CI, 95% of confidence interval; p, level of significance; vWF, von Willebrand factor.

**Supplementary Data 9**. Visual representation of adjusted association of coagulation proteins at ICU admission (baseline), end of follow-up and follow-up/baseline ratio with ICU length of stay. **Statistics:** data were calculated by Generalized Linear Models (GLM) with a gamma distribution. AMR, 95% confidence intervals (lower boundary: 2.5%, upper boundary: 97.5%), and p-value are shown. GLMs were adjusted by age, gender, levels of GOT and enoxaparin dosage during ICU stay at baseline point; by age, levels of GOT, enoxaparin dosage during ICU stay and anticoagulant therapy during follow-up, and by by age, levels of GOT and anticoagulant therapy at the end of follow-up. Significant associations are shown with an asterisk. **Abbreviations**: AMR, arithmetic mean ratio; aAMR, adjusted AMR; aPTT, activated partial thromboplastin time; INR, international normalized ratio; 95%CI, 95% of confidence interval; p, level of significance; vWF, von Willebrand factor.

**
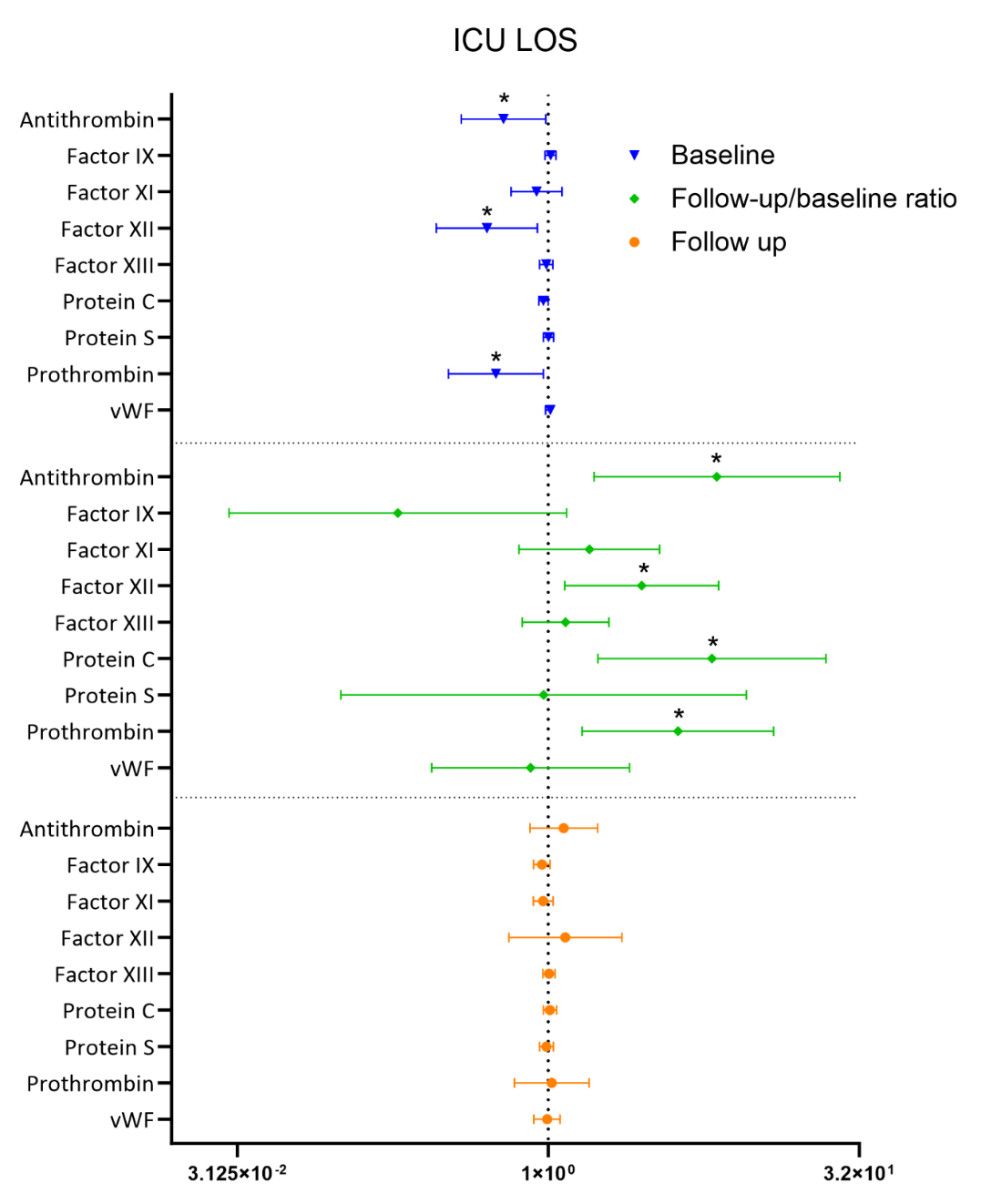
**

**Supplementary Data 10**. Association of significant coagulation proteins at ICU admission (baseline), end of follow-up and follow-up/baseline ratio with ICU LOS, stratifying by gender.

| Time point | Coagulation proteins | Males | | Females | |
| --- | --- | --- | --- | --- | --- |
|  |  | aOR (95%CI) | *p* | aOR (95%CI) | *p* |
| **Baseline** | Factor XII | 0.42 (0.2-0.88) | **0.028** | 0.74 (0.33-1.64) | 0.473 |
|  | Antithrombin | 0.55 (0.3-1.01) | 0.065 | 0.81 (0.35-1.91) | 0.648 |
|  | Prothrombin | 0.53 (0.25-1.09) | 0.095 | 0.47 (0.18-1.24) | 0.161 |
| **Follow-up/baseline ratio** | Factor XII | 3.33 (0.89-12.49) | 0.085 | 2.48 (0.77-7.97) | 0.171 |
|  | Antithrombin | 18.82 (2.6-136.39) | **0.007** | 3.12 (0.72-13.59) | 0.173 |
|  | Prothrombin | 7.71 (1.55-38.47) | **0.019** | 3.41 (0.83-13.97) | 0.132 |
|  | Protein C | 10.67 (2.36-48.23) | **0.005** | 1.44 (0.08-25.28) | 0.81 |
|  | INR | 0.05 (0.01-0.2) | **<0.001** | 45.34 (0.02-120002.19) | 0.397 |
| **End of the follow-up** | INR | 0.16 (0.04-0.62) | **0.014** | 0.52 (0.06-4.36) | 0.566 |

**Statistics**: data were calculated by Generalized Linear Models (GLM) with a gamma distribution. AMR, 95% confidence intervals (lower boundary: 2.5%, upper boundary: 97.5%), and p-value are shown, for both unadjusted and adjusted models. GLMs were adjusted by age, gender, levels of GOT and enoxaparin dosage during ICU stay at baseline point; by age, levels of GOT, enoxaparin dosage during ICU stay and anticoagulant therapy during follow-up, and by by age, levels of GOT and anticoagulant therapy at the end of follow-up. Significant associations are shown in bold. **Abbreviations**: AMR, arithmetic mean ratio; aAMR, adjusted AMR; INR, international normalized ratio; 95%CI, 95% of confidence interval; p, level of significance.
